# OnabotulinumtoxinA Displays Greater Biological Activity Compared to IncobotulinumtoxinA, Demonstrating Non-Interchangeability in Both In Vitro and In Vivo Assays

**DOI:** 10.3390/toxins12060393

**Published:** 2020-06-13

**Authors:** David Rupp, Greg Nicholson, David Canty, Joanne Wang, Catherine Rhéaume, Linh Le, Lance E. Steward, Mark Washburn, Birgitte P. Jacky, Ron S. Broide, Wolfgang G. Philipp-Dormston, Mitchell F. Brin, Amy Brideau-Andersen

**Affiliations:** 1 Allergan, an AbbVie company, Irvine, CA 92612, USA; Nicholson_Greg@Allergan.com (G.N.); Canty_David@Allergan.com (D.C.); juan_wang_2000@yahoo.com (J.W.); Rheaume_Catherine@Allergan.com (C.R.); Le_Linh@Allergan.com (L.L.); Steward_Lance@Allergan.com (L.E.S.); washburn_ms@yahoo.com (M.W.); Jacky_Birgitte@Allergan.com (B.P.J.); Broide_Ron@Allergan.com (R.S.B.); Brin_Mitchell@Allergan.com (M.F.B.); Brideau-Andersen_Amy@Allergan.com (A.B.-A.); 2 Faculty of Health, University Witten/Herdecke, 58455 Witten, Germany; wpd@haut-zentrum.com; 3 Department of Neurology, University of California, Irvine, Irvine, CA 92697, USA

**Keywords:** BOTOX, cell-based potency assay (CBPA), compound muscle action potential (CMAP) electrophysiology assay, digit abduction score (DAS) assay, dose conversion, interchangeability, light-chain activity high-performance liquid chromatograph (LCA-HPLC) assay, potency, Xeomin

## Abstract

Differences in botulinum neurotoxin manufacturing, formulation, and potency evaluation can impact dose and biological activity, which ultimately affect duration of action. The potency of different labeled vials of incobotulinumtoxinA (Xeomin^®^; 50 U, 100 U, or 200 U vials; incobotA) versus onabotulinumtoxinA (BOTOX^®^; 100 U vial; onabotA) were compared on a unit-to-unit basis to assess biological activity using in vitro (light-chain activity high-performance liquid chromatography (LCA-HPLC) and cell-based potency assay (CBPA)) and in vivo (rat compound muscle action potential (CMAP) and mouse digit abduction score (DAS)) assays. Using LCA-HPLC, incobotA units displayed approximately 54% of the protease activity of label-stated equivalent onabotA units. Lower potency, reflected by higher EC_50_, ID_50_, and ED_50_ values (pooled mean ± SEM), was displayed by incobotA compared to onabotA in the CBPA (EC_50_: incobotA 7.6 ± 0.7 U/mL; onabotA 5.9 ± 0.5 U/mL), CMAP (ID_50_: incobotA 0.078 ± 0.005 U/rat; onabotA 0.053 ± 0.004 U/rat), and DAS (ED_50_: incobotA 14.2 ± 0.5 U/kg; onabotA 8.7 ± 0.3 U/kg) assays. Lastly, in the DAS assay, onabotA had a longer duration of action compared to incobotA when dosed at label-stated equivalent units. In summary, onabotA consistently displayed greater biological activity than incobotA in two in vitro and two in vivo assays. Differences in the assay results do not support dose interchangeability between the two products.

## 1. Introduction

Botulinum neurotoxin serotype A (BoNT/A) is a highly potent toxin that causes muscle relaxation by inhibition of synaptic vesicle docking and fusion, thereby blocking acetylcholine release at neuromuscular junctions [1,2,3]. BoNT/A is produced by *Clostridium botulinum* Type A strain [4,5], which synthesizes a complex of a 150 kDa neurotoxin, along with a group of non-toxic neurotoxin-associated proteins (NAPs). Commercially available BoNT/A products are in the form of a complex with varying degrees of NAPs or only as the core 150 kDa neurotoxin devoid of NAPs.

Four BoNT/A products have been approved by the United States Food and Drug Administration (FDA) for a variety of therapeutic and cosmetic indications: onabotulinumtoxinA, approved in 1989 (BOTOX; Allergan, an AbbVie company, North Chicago, Illinois, USA; hereafter referred to as onabotA); abobotulinumtoxinA, approved in 2009 (DYSPORT^®^; Ipsen Ltd., Slough, UK; hereafter referred to as abobotA); incobotulinumtoxinA, approved in 2010 (Xeomin; Merz Pharmaceuticals; Frankfurt, Germany; hereafter referred to as incobotA); and prabotulinumtoxinA-xvfs, approved in 2019 (Jeuveau^®^; Evolus, Inc., Newport Beach, CA, USA; hereafter referred to as prabotA) [6]. These products are biological agents that are not chemically synthesized; as such, their compositions are structurally complex, having unique characteristics specific to each product that are key to their biological activity. Specifically, onabotA is formulated as an ~900 kDa BoNT/A complex [7,8], abobotA is formulated as an ~400 kDa BoNT/A complex [8,9,10], incobotA is formulated containing only the 150 kDa purified neurotoxin devoid of NAPs [9,11], and prabotA is formulated as a ~900 kDa BoNT/A complex [12]. Differences in how BoNT/A neuromodulators are manufactured, formulated, and evaluated for potency by specific assays may impact clinical features, such as dose, duration, efficacy, and immunogenicity [13]. Each manufacturer uses its own proprietary assay methods and reference standards to release drug product, which results in differences in unit doses that are not interchangeable [13]. To this end, abobotA and onabotA products contain label instructions with different dosing regimens for similar indications, supporting that product units are not interchangeable per product labelling. However, there is conflicting evidence regarding whether incobotA and onabotA units are interchangeable (at a 1:1 unit conversion ratio; [14,15,16,17,18,19,20,21]). Furthermore, European incobotA product labels (Xeomin and Bocouture^®^) now state “therapeutic equivalence” to onabotA for select indications [22,23]. However, other studies refute the claim of interchangeability between onabotA and incobotA (reviewed in [21,24,25,26]), and the discussion has also included preclinical testing [27,28,29,30,31,32].

The purpose of this study was to compare the potency of different labeled vials of incobotA (different toxin amounts/vial; 50 U, 100 U, and 200 U vials) versus onabotA (100 U vial) on a unit-to-unit basis to assess the biological activity of these products using both in vitro (light-chain activity high-performance liquid chromatography (LCA-HPLC) and cell-based potency assay (CBPA)) and in vivo (compound muscle action potential (CMAP) electrophysiology and digit abduction score (DAS)) assays. In agreement with previous findings [28,32], onabotA showed greater biological activity than incobotA in all assays, demonstrating that the potency units and performance of these two products in these pre-clinical assays are not interchangeable and each product should be administered as stipulated in product labeling.

## 2. Results

### 2.1. Light-Chain Activity High-Performance Liquid Chromatography (LCA-HPLC) Assay

**Figure 1:**
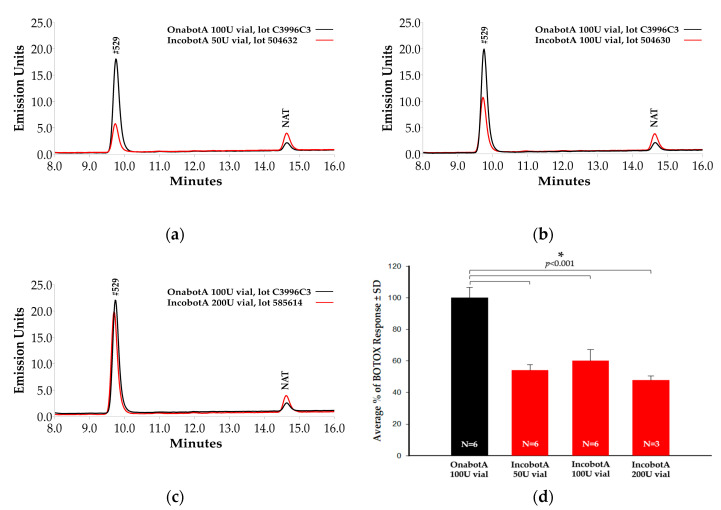
Light-chain activity high-performance liquid chromatography (LCA-HPLC) assay comparison of incobotA (50 U, 100 U, and 200 U vials) to onabotA (100 U vial). Representative chromatographs for (**a**) 50 U vial incobotA vs. 100 U vial onabotA, (**b**) 100 U vial incobotA vs. 100 U vial onabotA, and (**c**) 200 U vial incobotA vs. 100 U vial onabotA are shown. In the figure, #529 is the unquenched calibration peptide for SNAPtide 520 substrate for *C. botulinum* type A neurotoxin, and N-acetyl-tryptophan (NAT) is used as a stabilizer in human serum albumin. (**d**) Normalized data (SNAPtide cleavage product peak area (RFU)/labeled potency units, see methods for details) are demonstrated for onabotA (100 U vial) and all labeled vials of incobotA tested (50 U, 100 U, and 200 U vials). Normalized data are presented as mean ± SD. Differences in normalized means for incobotA (50 U, 100 U, and 200 U vials) versus onabotA (100 U vial) were analyzed using a one-way analysis of variance (ANOVA); * indicates a statistically significant difference of *p* < 0.05 vs. onabotA 100 U vial.

The LCA-HPLC assay measures SNAP-25 cleavage specificity [28]. In the context of this study, the light chain of BoNT/A cleaves a commercially available truncated BoNT/A substrate (SNAPtide^®^ 520, List Biological Laboratories, Inc., Campbell, CA, USA) derived from synaptosomal associated protein, 25 kDa (SNAP-25). Cleavage of SNAP-25, a soluble N-ethylmaleimide sensitive factor attachment receptor (SNARE), results in disruption of neuronal stimulation [3]. Light-chain protease activity has been shown to correlate with product potency (via mouse LD_50_ (mLD_50_)) and may be used to measure product stability as well, with atypical cleavage suggesting product instability [28]. Based on a product-labeled unit-to-unit comparison (Figure 1a–c), onabotA (100 U vial) showed greater cleavage of SNAP-25 substrate than incobotA (50 U, 100 U, and 200 U vials), as indicated by higher peaks at the #529 fragment area. Normalization of the ratio of SNAPtide cleavage product peak area (represented as relative fluorescence units; RFU) to labeled potency units demonstrates that light-chain protease activity was significantly lower for all labeled vials of incobotA (50 U: 54.0 ± 3.5%, mean ± standard deviation (SD); 100 U: 60.0 ± 7.2%; 200 U: 48 ± 2.8%) compared to onabotA (100 U: 100.0 ± 6.7%; all comparisons, *p* < 0.001), resulting in approximately 54% of predicted values based on product vial size at equivalent stated units (Figure 1d). No atypical cleavage product, as previously reported for incobotA [28], was observed in any of the onabotA (100 U) or incobotA (50 U, 100 U, and 200 U vials) product vials tested.

### 2.2. Cell-Based Potency Assay (CBPA)

**Figure 2:**
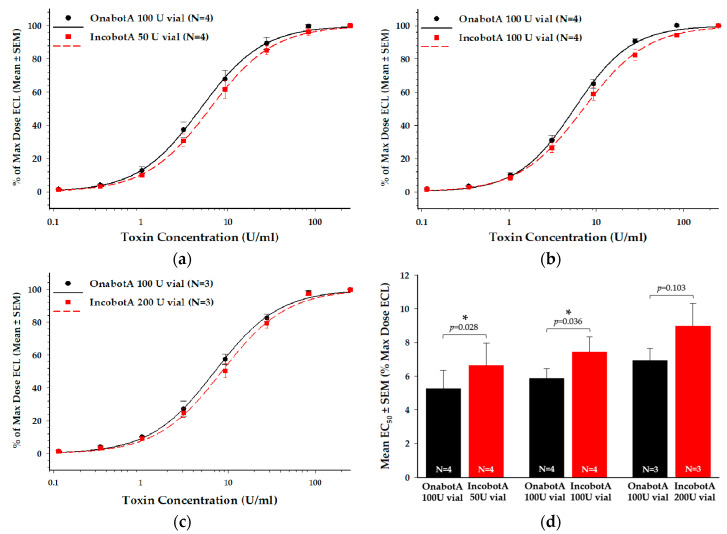
Cell-based potency assay (CBPA) comparison of incobotA (50 U, 100 U, and 200 U vials) to onabotA (100 U vial). Mean dose response curves for (**a**) 50 U vial incobotA vs. 100 U vial onabotA, (**b**) 100 U vial incobotA vs. 100 U vial onabotA, and (**c**) 200 U vial incobotA vs. 100 U vial onabotA are shown as percent of max dose ECL ± standard error of the mean (SEM). Data for dose-response curves represent N = 3 or N = 4 replicate assays for each comparison. (**d**) Mean EC_50_ values ± SEM for each comparison are shown. Dose response curves were assessed using regression analyses and mean product EC_50_ values were compared using a two-tailed paired *t*-test; * indicates a statistically significant difference of *p* < 0.05 vs. onabotA 100 U vial.

The SNAP-25_197_ SiMa H1 electrochemiluminescent (ECL) CBPA is an in vitro cell-based assay that measures the key steps of BoNT/A intoxication: receptor-mediated cell binding and internalization, translocation of the protease domain (light chain) into the cytosol, and proteolytic cleavage of SNAP-25, allowing direct comparison of BoNT/A product biological activity in vitro [33]. Based on a product-labeled unit-to-unit comparison (Figure 2a–c), each labeled vial of incobotA tested (50 U, 100 U, and 200 U vials) demonstrated reduced relative potency in the CBPA, as indicated by a rightward shift in the dose response curves compared to the respective onabotA (100 U vial) reference data. This reduced potency resulted in a 1.3-fold difference in incobotA compared to onabotA in the CBPA (Figure 2d; Table 1), which was statistically significant for the 50 U (*p* = 0.028) and 100 U (*p* = 0.036) vials, but not the 200 U vial (*p* = 0.103).

### 2.3. Compound Muscle Action Potential (CMAP) Electrophysiology Assay

**Figure 3:**
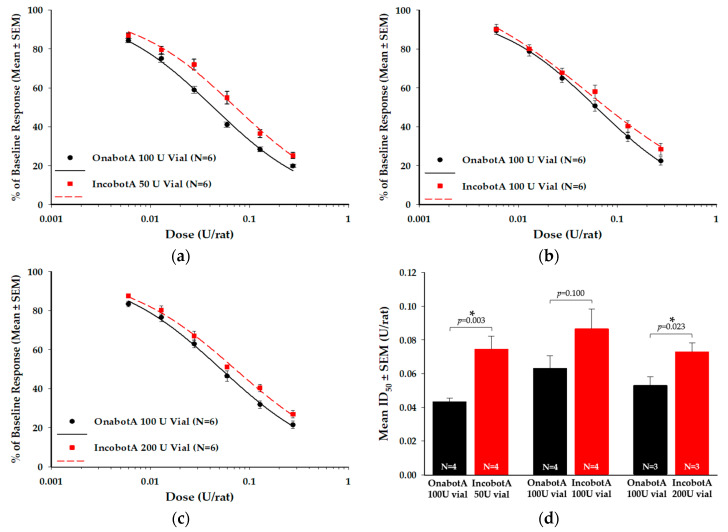
Compound muscle action potential (CMAP) electrophysiology assay comparison of incobotA (50 U, 100 U, and 200 U vials) to onabotA (100 U vial). Representative dose-response curves for (**a**) 50 U vial incobotA vs. 100 U vial onabotA, (**b**) 100 U vial incobotA vs. 100 U vial onabotA, and (**c**) 200 U vial incobotA vs. 100 U vial onabotA are shown as percent of baseline response ± SEM. Data for dose-response curves represent N = 5 animals per dose, which was replicated six times for each product. (**d**) Mean ID_50_ values ± SEM for each comparison are shown. Dose response data were assessed using regression analyses and mean product ID_50_ values were individually compared using a one-way ANOVA; * indicates a statistically significant difference of *p* < 0.05 vs. onabotA 100 U vial.

The CMAP is an in vivo electrophysiological assay that quantitatively measures synaptic transmission at the neuromuscular junction following BoNT/A intramuscular (IM) injection [34]. In this study, the CMAP assay evaluated the potency of the injected BoNT/A based on dose-dependent decrease in action potential amplitude of local muscle fibers in the hind limb [34]. Based on a product-labeled unit-to-unit comparison (Figure 3a–c), each labeled vial of incobotA tested (50 U, 100 U, and 200 U vials) demonstrated reduced relative potency in the CMAP assay, as indicated by a rightward shift in the dose response curves compared to the respective onabotA reference (100 U vial). This reduced potency resulted in a 1.4−1.7-fold difference in incobotA compared to onabotA in the CMAP assay (Figure 3d; Table 1), which was statistically significant for both the 50 U vial (*p* = 0.003) and 200 U vial (*p* = 0.023), but not the 100 U vial (*p* = 0.100).

### 2.4. Digit Abduction Score (DAS) Assay

**Figure 4:**
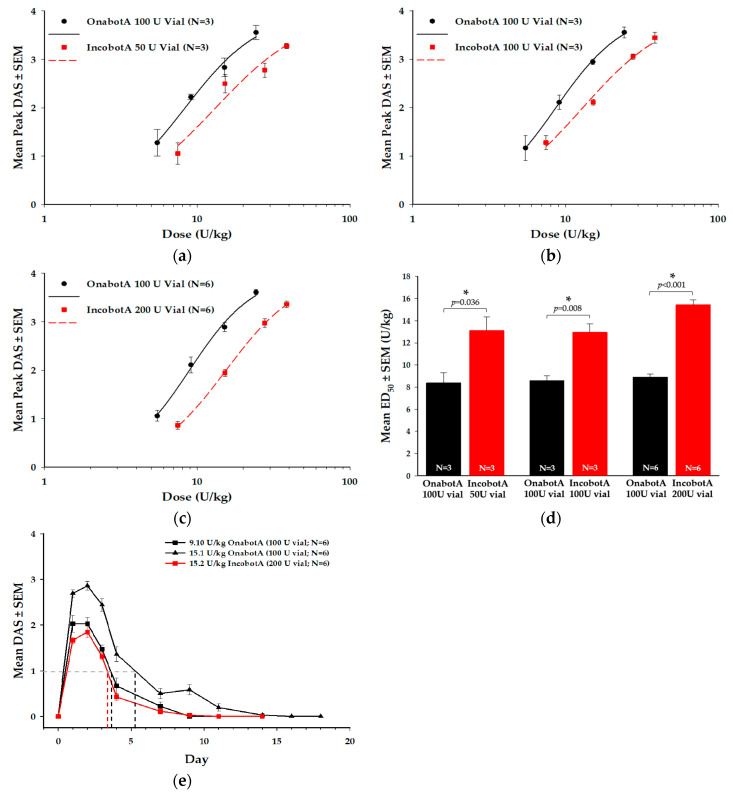
Digit abduction score (DAS) assay comparison of different labeled vials of incobotA (50 U, 100 U, and 200 U vials) to onabotA (100 U vial). Mean peak DAS dose-response curves (± SEM) are shown for (**a**) 50 U vial incobotA vs. 100 U vial onabotA, (**b**) 100 U vial incobotA vs. 100 U vial onabotA, and (**c**) 200 U vial incobotA vs. 100 U vial onabotA. Data for dose-response curves represent N = 6 animals per dose, which was replicated three to six times for each product. (**d**) Mean ED_50_ values (± SEM) for each comparison are shown. (**e**) Mean DAS responses over time and mean time to return-to-ED_25_ (represented by dotted lines) for 9.10 U.kg onabotA (100 U vial), 15.1 U/kg onabotA (100 U vial), and 15.2 U/kg of incobotA (200 U vial) are shown. Mean peak DAS responses were assessed using regression analyses, while mean ED_50_ values were compared using a one-way ANOVA and mean return-to-ED25 values compared using a Kruskal-Wallis ANOVA; * indicates a statistically significant difference of *p* < 0.05 vs. onabotA 100 U vial.

The DAS assay is an in vivo assessment of toxin-induced muscle paralysis following injection of BoNT/A toxin into the hind limb muscle of a rodent. The DAS assay can be used to compare the potency of different BoNT/A products on muscle paralysis, as well as the duration of action [35,36]. In this assay, all labeled vials of incobotA tested (50 U, 100 U, and 200 U vials) showed reduced relative potency compared to onabotA (100 U vial), as indicated by a rightward shift in the dose-response curves for the mean peak DAS response (Figure 4a–c). This reduced potency resulted in a 1.5−1.7-fold difference in incobotA compared to onabotA (Figure 4d; Table 1), which was statistically significant for the 50 U (*p* = 0.036), 100 U (*p* = 0.008), and 200 U (*p* < 0.001) vials. This difference in potency translated into a longer duration of muscle paralysis for onabotA compared to incobotA at comparable doses based on labeled units. As shown in the representative example (Figure 4e), when administered at comparable doses based on labeled units (~15 U/kg), onabotA demonstrated a greater DAS response and longer duration of action than incobotA. Moreover, a lower dose of onabotA (9.1 U/kg) showed a similar DAS response and duration as the higher dose of incobotA (15.2 U/kg). The calculated area-under-the-curve (AUC) value for 15.1 U/kg onabotA was significantly higher (13.7 ± 1.2 (mean ± SEM)) compared to both 15.2 U/kg incobotA (6.0 ± 0.5; *p* < 0.001) and 9.1 U/kg onabotA (7.4 ± 0.5; *p* < 0.001), indicating that more incobotA was required to achieve comparable peak response and duration as onabotA in the DAS assay. Mean AUC values for 9.1 U/kg onabotA and 15.2 U/kg incobotA did not differ statistically (*p* > 0.05). Furthermore, the mean (± SEM) time to return-to-ED_25_ for 15.1 U/kg onabotA was significantly greater than 15.2 U/kg incobotA (5.2 ± 0.5 days vs. 3.4 ± 0.1 days, respectively; *p* = 0.008). However, 15.2 U/kg incobotA did not differ from 9.10 U/kg onabotA (3.8 ± 0.2 days; *p* = 0.526).

## 3. Discussion

Since the introduction of incobotA (Merz Pharmaceuticals received FDA approval in 2010), there has been on-going debate within the literature as to the interchangeability of incobotA and onabotA at a uniform clinical dose conversion (1:1) ratio across a variety of indications (reviewed in [19,20,26,37,38]). Many studies refute the interchangeability of these products [24,25,39,40,41,42] and highlight that differences in injection volumes, patterns, and techniques, as well as distribution/diffusion patterns and other factors, vary between BoNT/A products [26]. For example, Moers-Carpi et al. found that 20 U of onabotA was as effective as 30 U of incobotA at treating glabellar lines in a randomized, double-blind study [24], demonstrating the non-interchangeability of units of onabotA and incobotA in this indication. Additionally, Yeilding and Fezza concluded that although both incobotA and onabotA products were safe and effective at reducing facial wrinkles, onabotA had statistically greater long-term efficacy, as measured at multiple timepoints (up to 4 months) following the initial injection [25].

In contrast, several clinical studies support the interchangeability of incobotA and onabotA, suggesting that both products have similar clinical potencies, biological activity, and duration of effect (as reviewed in [19,20,37,38,43]). For example, Benecke et al. reported similar efficacy and safety profiles for NT 201 (incobotA) for the treatment of cervical dystonia at 4 and 16 weeks to that observed for onabotA [44] and Sattler et al. concluded that incobotA was equally effective as onabotA in the treatment of glabellar frown lines at 4 and 12 weeks post-treatment [17]. However, as reviewed by Brin et al. [13], the non-inferiority design and lack of intermediate time points limit the types of conclusions that can be drawn from these two studies. Additionally, a randomized double-blind study by Rappl et al. found that incobotA demonstrated a faster onset and longer duration of effect than onabotA (at a 1:1 dose ratio) and abobotA (at a 1:3 dose ratio) [16]. However, these data conflict with previous publications, as acknowledged by the authors, and the reconstitution and injection procedures used in this study differ from instructions stated in the incobotA [45], onabotA [46], and abobotA [47] product inserts, resulting in the administration of off-label doses. Finally, Kane et al. found that onabotA and incobotA were equivalent at the 20 U dose for the treatment of glabellar frown lines at 1−4 months post-injection [18]. However, a low responder threshold (1-grade change on the Facial Wrinkle Scale (FWS)) may have contributed to the high response rates observed in this study [18], providing little margin within which to detect treatment differences between incobotA and onabotA (i.e., a ceiling effect) [13].

The debate on the interchangeability of onabotA and incobotA products has also included preclinical testing [27,28,29,30,31,32]. An in vivo study found that incobotA had lower potency than indicated on the product label (100 U vial), with LD_50_ values ranging from 69−78 U in mice [29]. Reduced potency of incobotA compared to onabotA was later confirmed in a follow-up study using the DAS assay [27]. Furthermore, hind limb paresis following onabotA, abobotA, or incobotA injection was assessed by analyzing the running-wheel performance of mice [32]. In this study, incobotA demonstrated reduced potency relative to onabotA, where the authors determined a ratio of 0.75:1 to 0.50:1 of incobotA to onabotA achieved equivalent potency, confirming that these products are not interchangeable [32]. In contrast, Frevert [30] found a higher mean concentration of BoNT/A neurotoxin in onabotA compared to incobotA using sandwich enzyme-linked immunosorbent assay (ELISA). In this paper, the authors emphasized nominal labeled units, but failed to test biological activity of the products (e.g., via DAS, mLD_50_, or CBPA). Dressler et al. [31] found no differences between onabotA and incobotA potency labeling using an mLD_50_ assay, concluding a 1:1 conversion ratio between the two products. However, as noted previously by Brin et al. [13], Merz adds human serum albumin (HSA) as a stabilizer to its undisclosed diluent [31] and stabilizers have resulted in enhanced BoNT/A activity in the mLD_50_ assay [48].

Hunt et al. [28] previously detected an atypical SNAPtide cleavage product in incobotA (100 U vial) that was not observed in either onabotA (100 U vial) or abobotA (500 U vial). The authors considered that this atypical SNAPtide cleavage product may be caused by damaged toxin or a contaminating protease that cleaves SNAPtide 520. This secondary cleavage product was not detected in the current study using the same assay and conditions as described therein [28], suggesting that incobotA product has changed over time. However, despite elimination of this secondary cleavage product, the biological activity of incobotA in the present study was lower than onabotA (100 U vial) when tested on a unit-to-unit basis at all labeled vials (incobotA 50 U, 100 U, or 200 U vials) in multiple preclinical assays.

It is important to highlight several caveats that must be considered when reviewing comparative BoNT/A product studies. These include: manufacturing process and final formulation/composition (e.g., purification method or finishing procedure, the presence or absence of NAPs), experimental design (e.g., non-inferiority trials vs. equivalence studies; number of data points collected; BoNT/A product doses tested), biological assay parameters (e.g., strain of animal used, timing of data collection, housing conditions), which can all impact the results and conclusions of these comparative product studies (reviewed in [13]).

With these caveats in mind, and in agreement with previous findings [27,28], the current study found on a unit-to-unit basis, onabotA (100 U vial) consistently displayed greater biological activity than all labeled vials of incobotA tested (50 U, 100 U, and 200 U vials) in two in vitro and two in vivo assays. Combined, these data demonstrate that labeled units of onabotA and incobotA are not interchangeable. As differences in biological activity and product potency may affect clinical outcomes, BoNT/A products should be administered as stipulated in product labeling.

## 4. Materials and Methods

### 4.1. BoNT/A Products

In vitro (LCA-HPLC and CBPA) and in vivo (CMAP and DAS) assays were utilized to assess the potency of labeled vials of incobotA (50 U, 100 U, and 200 U vials) versus onabotA (100 U vial) on a unit-to-unit basis. As summarized in Brin et al. [13], onabotA and incobotA have different manufacturing processes and formulations. Specifically, onabotA is purified using crystallization and is formulated as an ~900 kDa BoNT/A complex protein. Each 100 U vial of onabotA contains 0.5 mg of human serum albumin and 0.9 mg of sodium chloride as formulation excipients. For finishing, onabotA is vacuum dried. In contrast, incobotA is purified using chromatography and is formulated containing the 150 kDa BoNT/A protein only. All labeled vials of incobotA (50 U, 100 U, and 200 U) contain 1 mg of human serum albumin and 4.7 mg of sucrose as formulation excipients. For finishing, incobotA is lyophilized. Product lot numbers are provided for each assay below.

### 4.2. Light-Chain Activity High-Performance Liquid Chromatography (LCA-HPLC) Assay

BoNT/A products (incobotA 50 U (Lot #504632 and 598468), 100 U (Lot #504630 and 517048), 200 U (Lot #585614) vials and onabotA 100 U (Lot #C3982C3 an C3996C3) vial) were tested in triplicate using the LCA-HPLC assay (see Hunt et al. for full methodological details [28]). Product vials were reconstituted on the day of testing with 1 mL of digestion buffer (0.5 mM zinc acetate, 2 mM dithiothreitol, 0.05% Tween 20 in 50 mM HEPES, pH 7.4). Aliquots (350 µL) of each reconstituted sample were transferred to separate reaction tubes and heated at 37 °C for 30 min (reduction step). Next, 25 μL of 200 μM SNAPtide 520 (List Biological Laboratories Inc., Campbell, CA, USA) was added to each reaction tube (equivalent to 13.3 μM SNAPtide 520) followed by incubation at 30 °C for 20 h (digestion step). Reactions were quenched following the addition of 25 μL of 5% trifluoroacetic acid (TFA). The contents of each tube were then transferred to HPLC vials for analysis. The fluorescently-labeled cleavage product(s) were separated and detected via a reverse-phase (RP)-HPLC method [28] using a Waters 2695 XE Separations Module and a Waters 2475 Multi λ Fluorescence Detector (Waters Corp., Milford, MA, USA). In brief, 25 µL of cleavage products were loaded onto a Symmetry C18 column (300 Å, 3.5 µm, 4.6 mm × 150 mm; Waters Corp.), maintained at 35 °C. Separation was accomplished using a 10−90% CH_3_CN (0.1% TFA) gradient for 30 min, with a flow-rate of 1 mL/min. The column effluent was monitored fluorescently (excitation λ = 322 nm, emission λ = 420 nm) to detect the o-aminobenzoic acid fluorophore on the N-terminal cleaved fragment of SNAPtide 520. SNAPtide cleavage product (#529, List Biological Laboratories, Inc.) was utilized to identify retention time of the cleavage product, as #529 is the unquenched calibration peptide for SNAPtide 520 substrate for *C. botulinum* type A neurotoxin.

In total, two lots of onabotA (100 U vial) were evaluated against two (50 U vial and 100 U vial) or one (200 U vial) lot of incobotA, with three vials tested from each lot. Testing was performed on two separate test days and data were pooled for analysis. Data were collected and analyzed via Waters Empower Pro software (Waters Corp.). Subsequent data were normalized by calculating the ratio of SNAPtide cleavage product peak area (RFU) to labeled potency units. Data were then expressed as a percentage of the average normalized onabotA ratio. Calculated average ratio values were compared for statistical significance via one-way ANOVA. Post-hoc comparisons versus onabotA (control group) were determined using the Holm-Sidak method. All plotting of normalized data and statistical analyses were carried out using SigmaPlot (SysStat Software, Inc., v13.0; San Jose, CA, USA).

### 4.3. Cell-Based Potency Assay (CBPA)

The methodology for the research version of the CBPA used in this study was adapted from Fernández-Salas et al. [33]. To summarize, human neuroblastoma SiMa H1 cells (DSMZ; Braunschweig, Germany) were plated onto poly-D-lysine (PDL) 96-well plates at 100,000 cells/well (optimized seeding cell density for Allergan’s CBPA [33]) in serum-free media (SFM) with 25 µg/mL of GT_1b_ (Enzo Life Sciences; Farmingdale, NY, USA) for three days. BoNT/A products (incobotA 50 U (Lot #598468), 100 U (Lot #517048), 200 U (Lot #524231) vials and onabotA 100 U (Lot #C3995C3 and C3996C3) vial) were reconstituted in SFM with 25 µg/mL of GT1_b_ on the day of testing and each product was tested at a dose range of 0.04−250 U/mL, with each dose run in triplicate. IncobotA and onabotA were run head-to-head on the same assay plate to allow for direct comparison of product potencies. After 24 h of treatment, the BoNT/A toxin was removed, and cells were incubated in fresh SFM with 25 µg/mL of GT1_b_ for an additional 48 h. Detection of cleavage product was determined using an ELISA-based method [33]. In brief, cells were lysed and lysates were transferred to MSD High Bind plates (Meso Scale Diagnostics, LLC, Rockville, MD, USA) coated with anti-SNAP-25_197_ mAb 2E2A6. Plates were then washed with 0.05% PBST and incubated with SULFO-TAG NHS-Ester (Meso Scale Diagnostics, LLC) labeled detection pAb anti-SNAP-25 (Sigma, St. Louis, MO, USA). Captured, BoNT/A toxin-cleaved SNAP-25 was then quantitated on an MSD plate reader (SECTOR S 600, Meso Scale Diagnostics, LLC). In total, two lots of onabotA (100 U vial) and one lot each of incobotA (50 U vial, 100 U vial, and 200 U vial) were evaluated.

To account for plate-to-plate variability, ECL data from each product on a given assay plate were normalized to the maximal dose values for that product to obtain percent of max dose values (% of Max Dose ECL). Data were analyzed using non-linear regression (3-parameter logistic model applying curve constraint at the top equal to 100) to generate pharmacological potency (EC_50_) values using GraphPad Prism (GraphPad Software Inc., San Diego, CA, USA, v7.02). To determine whether potency was significantly different between the two products, mean EC_50_ values for onabotA and incobotA lots that were run head-to-head on the same assay plates were compared via paired two-tailed t-test. In addition, the individual potency values from onabotA and incobotA were used to generate average relative potency ratios of incobotA to onabotA. These values represent the fold increase in incobotA required to obtain the same observed potency as that observed for onabotA in the CBPA assay. All plotting, curve fitting, and statistical analyses were carried out using GraphPad Prism.

### 4.4. Compound Muscle Action Potential (CMAP) Electrophysiology Assay

All procedures were approved by Allergan’s Animal Care and Use Committee (AACUC; protocol# 731-100014-2016; approved 22 June 2016). Methodological details for the CMAP electrophysiology assay were adapted from published sources [34,49]. Eight-week-old (221.2 ± 8.2 g) female Sprague-Dawley rats (Charles River, Hollister, CA, USA) were used in this study. Female rats are routinely used in our CMAP assay to provide standardization and published CMAP data are generally derived from females (e.g., [34,50,51]). Rats were socially housed in groups of four in shoebox cages, with woodchip shavings, and cardboard tunnels. Rats were maintained on a 12-h light-dark cycle, with food and water provided ad libitum. Electrophysiological measurements were performed at baseline (day 0) and following BoNT/A toxin treatment (day 3). Two separate manufactured lots of onabotA (100 U (Lot # C3995C3 and C3996C3) vial) were tested head-to-head against two separate lots each of different labeled vials of incobotA (50 U (Lot #504632 and 598468), 100 U (Lot #506807 and 517048), or 200 U (Lot #524231 and 585614) vials) in triplicate, totaling 18 test days, with a new vial of each product being reconstituted each test day. BoNT/A products were diluted in 0.9% saline to yield the following final dose concentrations for each: 0.0060, 0.0129, 0.0277, 0.0595, 0.128, or 0.275 U of toxin delivered per animal. On each test day, n = 5 weight-matched rats were tested per dose of BoNT/A product. A 50 µL volume of diluted toxin was injected IM into the medial head of the gastrocnemius (calf) muscle with sterile 2.5 mL glass syringes (Hamilton Co.; Reno, NV, USA) affixed to PB600-1 repeating dispensers (Hamilton Co.). Electrophysiological measurements were performed by inserting a recording electrode into the gastrocnemius muscle, a reference electrode parallel to the distal tendon of the same muscle, and by placing a ground electrode between these two points. At baseline, rats were tattooed at the site of the recording electrode to ensure that post-toxin measurements were recorded at the same anatomical location. The needle recording electrodes were partially covered by PE tubing to ensure that the same recording depth was used for each rat. Constant current from a bipolar stimulating electrode was used to transdermally excite the sciatic nerve during recordings at a frequency of 1 Hz. The CMAP response associated with each peak mean of 3 sets of 10 responses to a supramaximal stimulus was digitized and recorded. Toxin potency/activity is represented as the inhibition of CMAP responses between the baseline and post-toxin readings.

For individual replicates, scatterplots of the percent of the CMAP baseline responses versus each toxin dose (U/rat) were generated and regression analyses were carried out via a 3-parameter logistic model for each replicate applying curve restraints at the maximum (100) and minimum (0) values. In addition, the regression analysis output parameters were used to calculate inhibitory dose-50 (ID_50_) values for each product. Individual replicate ID_50_ values were used to calculate a mean (± SEM) ID_50_ value for each product as well. Prior to the pooling of any data, mean ID_50_ values were compared across replicate sets via one-way ANOVA to ensure they did not differ significantly. To determine whether there was a significant potency difference between the two products, mean ID_50_ values for onabotA (100 U vial) and incobotA (50, 100, or 200 U vials) lots that were run head-to-head on the same experimental days (using the pooled data for the two lots of each product) were compared via one-way ANOVA. An additional comparison was carried out between the three incobotA mean ID_50_ values (pooled within the individual labeled vials) versus the single pooled onabotA mean ID_50_ values (all replicates; N = 18) using a one-way ANOVA with Holm-Sidak post-hoc multiple comparisons versus onabotA (control group). In addition, the individual potency values from onabotA and incobotA samples tested head-to-head were used to generate average relative potency ratios of incobotA to onabotA. All plotting, curve fitting, and statistical analyses were carried out using SigmaPlot.

### 4.5. Digit Abduction Score (DAS) Assay

All procedures were approved by AACUC (protocol# 225-100051-2016; approved 5 February 5 2016). Full methodological details for the DAS assay are provided in [35,36]. In summary, female CD-1 mice (Charles River), with a weight range of 20−28 g, and the weight of each mouse within an experimental replicate standardized to be within ± 2 g of the group mean, were used in this study. Similar to the reasons provided for the CMAP assay, female mice are routinely used in our DAS assay to provide standardization and published mouse DAS data are generally derived from females (e.g., [35,51,52]). Mice were housed in groups of six in polycarbonate cages with Alpha-dri^®^ bedding, shredded paper nesting material, and a plastic dome. Mice were maintained on a 12-h light-dark cycle, with food and water provided ad libitum. BoNT/A products (incobotA 50 U (Lot #598468), 100 U (Lot #517048), 200 U (Lot #524231 and 585614) vials and onabotA 100 U (Lot # C3982C3, C3995C3, C3996C3, and C4242C3) vial) were tested head-to-head in triplicate, with each replicate commencing on six different start days, and a new vial of each product being reconstituted for each replicate. The comparison of onabotA 100 U vial vs. incobotA 200 U vial was tested for an additional 3 replicates (totaling 6 replicates). For each experimental replicate, four doses of each BoNT/A product were tested, with n = 6 mice per dose. Working solutions for onabotA and incobotA were prepared via serial dilution in 0.9% saline to yield the following final concentrations for each: 24.3, 15.1, 9.10, and 5.47 U/kg for onabotA; 38.5, 27.7, 15.2, and 7.46 U/kg for incobotA. These doses represent the projected ED_25_, ED_50_, ED_75_, and ED_90_ values for each product based on previous assays with the current or related lots [27]. All dosing solutions were randomized into sterile 250 µL glass syringes (Hamilton Co.) affixed to PB600-1 repeating dispensers (Hamilton Co.). On day 0, mice were given a 5 µL IM injection of the appropriate diluted test product into the right gastrocnemius muscle using a 30 gauge, half-inch needle. Toxin-induced muscle paralysis was scored using the 5-point DAS scale (scored 0–4), where a score of “0” represents a normal response and a score of “4” represents maximum reduction in digit abduction [35,36]. All groups were rated for DAS, as well as observed for any changes in general activity level within the home cage according to AACUC-approved criteria for assessing the clinical well-being of a test animal, on days 1−4, 7, 9, 11, 14, 16, 18, 21, 23, 25, 28, and 30. However, if all mice in the group were scored as DAS = 0 for two consecutive observation days, DAS rating and observation of general activity ceased.

For individual replicates, scatterplots of the maximum average DAS versus dose (U/kg) were generated and regression analyses were carried out via a 3-parameter logistic model for each replicate applying curve restraints at the maximum (4) and minimum (0) values. In addition, the regression analysis output parameters were used to calculate ED_50_ values for each product. Individual replicate ED_50_ values were used to calculate a mean (± SEM) ED_50_ value for each product as well. Prior to pooling any of the data, mean ED_50_ values were compared across replicate sets via one-way ANOVA to ensure they did not different significantly. To determine whether mean values were significantly different between the two products, mean ED_50_ values for onabotA and incobotA lots that were run head-to-head on the same experimental days (using the pooled data where applicable) were compared via one-way ANOVA. An additional comparison was carried out with the three incobotA mean ED_50_ values (pooled within the individual labeled vials) versus the single pooled onabotA mean ED_50_ value (all replicates; N = 12) using a one-way ANOVA with Holm-Sidak post-hoc multiple comparisons versus onabotA (control group). In addition, the individual potency values from onabotA and incobotA samples tested head-to-head were used to generate average relative potency ratios of incobotA to onabotA. Lastly, for comparison of duration of action, line and scatter plots of ‘DAS versus time (day)’ were determined and AUC values were calculated for the duration plots for both onabotA and incobotA presented in Figure 4e. Individual replicate AUC values were used to calculate a mean (± SEM) AUC value, which were compared via one-way ANOVA. Time (days) to return-to-ED_25_ were calculated from the intersection of the duration offset lines with DAS = 1 (ED_25_) on the appropriate graphs for each of the six replicates for the three dose groups. Mean return-to-ED_25_ values were compared via Kruskal-Wallis ANOVA on ranks using Tukey’s post-hoc tests. All plotting, curve fitting, and statistical analyses were carried out using SigmaPlot.

## Figures and Tables

**Table 1:** In vitro (CBPA) and in vivo (CMAP and DAS) comparison of onabotA (100 U vial) versus different labeled vials of incobotA (50 U, 100 U, and 200 U vials).

Product	CBPA EC_50_ (U/mL) (Mean ± SEM)	CBPA Fold-Difference ^3^ (Mean ± SD)	CMAP ID_50_ (U/rat) (Mean ± SEM)	CMAP Fold-Difference ^4^ (Mean ± SD)	DAS ED_50_ (U/kg) (Mean ± SEM)	DAS Fold-Difference ^4^ (Mean ± SD)
OnabotA (100 U vial) ^1^	5.3 ± 1.1	-	0.043 ± 0.002	-	8.4 ± 0.9	-
IncobotA (50 U vial) ^1^	6.6 ± 1.3 *	1.3 ± 0.1	0.075 ± 0.008 *	1.7 ± 0.2	13.1 ± 1.2 *	1.6 ± 0.4
OnabotA (100 U vial) ^1^	5.9 ± 0.6	-	0.063 ± 0.007	-	8.6 ± 0.4	-
IncobotA (100 U vial) ^1^	7.4 ± 0.9 *	1.3 ± 0.1	0.087 ± 0.012	1.4 ± 0.1	13.0 ± 0.8 *	1.5 ± 0.1
OnabotA (100 U vial) ^1^	6.9 ± 0.7	-	0.053 ± 0.005	-	8.9 ± 0.3	-
IncobotA (200 U vial) ^1^	9.0 ± 1.3	1.3 ± 0.1	0.073 ± 0.005 *	1.4 ± 0.1	15.4 ± 0.5 *	1.7 ± 0.2
Pooled onabotA (100 U vials) ^2^	5.9 ± 0.5	-	0.053 ± 0.004	-	8.7 ± 0.3	-
Pooled incobotA (50 U, 100 U, and 200 U vials) ^2^	7.6 ± 0.7	1.3	0.078 ± 0.005	1.5 ± 0.3	14.2 ± 0.5	1.7 ± 0.2

^1^ Data shown are pooled by lot per product and unit vial. ^2^ Data shown are pooled by product across all lots utilized and unit vials. Sample sizes for individual assays are specified in the methods. ^3^ Calculated as an average of the ratios of incobotA (50, 100, or 200 U vials) EC_50_ to onabotA (100 U vial) EC_50_ for samples run head-to-head on the same assay plate. ^4^ Calculated relative to onabotA (100 U vial). * Indicates a statistically significant difference at *p* < 0.05 vs onabotA 100 U vial based on two-tailed paired t-test (CBPA) or one-way ANOVA with Holm-Sidak post-hoc analysis (CMAP and DAS).
